# Clinical Significance and the Role of Guanylate-Binding Protein 5 in Oral Squamous Cell Carcinoma

**DOI:** 10.3390/cancers13164043

**Published:** 2021-08-11

**Authors:** Pei-Feng Liu, Chih-Wen Shu, Cheng-Hsin Lee, Huei-Cin Sie, Huei-Han Liou, Jiin-Tsuey Cheng, Luo-Ping Ger, Chun-Lin Chen, Chien-Chou Chen, Chun-Feng Chen

**Affiliations:** 1 Department of Biomedical Science and Environmental Biology, College of Life Science, Kaohsiung Medical University, Kaohsiung 80708, Taiwan; pfliu@kmu.edu.tw (P.-F.L.); or R980084@kmu.edu.tw (C.-H.L.); 2 Department of Medical Research, Kaohsiung Medical University Hospital, Kaohsiung 80708, Taiwan; 3 Center for Cancer Research, Kaohsiung Medical University, Kaohsiung 80708, Taiwan; 4 Institute of Biomedical Sciences, National Sun Yat-sen University, Kaohsiung 80424, Taiwan; 5 Institute of BioPharmaceutical Sciences, National Sun Yat-sen University, Kaohsiung 80424, Taiwan; cwshu@g-mail.nsysu.edu.tw; 6 Department of Pathology and Laboratory Medicine, Kaohsiung Veterans General Hospital, Kaohsiung 81362, Taiwan; minikiki@vghks.gov.tw; 7 Department of Medical Education and Research, Kaohsiung Veterans General Hospital, Kaohsiung 81362, Taiwan or hhliou@vghks.gov.tw (H.-H.L.); lpger0329@gmail.com (L.-P.G.); 8 Department of Biological Sciences, National Sun Yat-sen University, Kaohsiung 80424, Taiwan; tusya@mail.nsysu.edu.tw (J.-T.C.); chunlinchen@mail.nsysu.edu.tw (C.-L.C.); 9 Family Medicine Division, Zuoying Branch of Kaohsiung Armed Forces General Hospital, Kaohsiung 81342, Taiwan; 10 Department of Stomatology, Kaohsiung Veterans General Hospital, Kaohsiung 81362, Taiwan; 11 Department of Dental Technology, Shu-Zen Junior College of Medicine and Management, Kaohsiung 82144, Taiwan; 12 School of Dentistry, Kaohsiung Medical University, Kaohsiung 80708, Taiwan

**Keywords:** oral squamous cell carcinoma, guanylate binding protein 5, malignancy, prognosis

## Abstract

**Simple Summary:**

The survival rate of oral squamous cell carcinoma (OSCC) patients has not been improved in the past few decades, likely as a result of a lack of therapeutic targets. Through next generation sequencing for tumor tissues of OSCC patients, the gene expression level of guanylate binding protein 5(GBP5) was significantly elevated in tumor tissues compared with adjacent normal tissues and associated with poor prognosis in OSCC patients. Moreover, we found that GBP5 modulated cell cycle, invasion/migration, and cancer stemness in OSCC cells. Our study indicated that GBP5 might be a potential biomarker and therapeutic target for OSCC patients.

**Abstract:**

Guanylate binding protein 5 (GBP5) is the interferon (IFN)-inducible subfamily of guanosine triphosphatases (GTPases) and is involved in pathogen defense. However, the role played by GBP5 in cancer development, especially in oral squamous cell carcinoma (OSCC), is still unknown. Herein, next-generation sequencing analysis showed that the gene expression levels of GBP5 were significantly higher in OSCC tissues compared with those found in corresponding tumor adjacent normal tissues (CTAN) from two pairs of OSCC patients. Higher gene expression levels of GBP5 were also found in tumor tissues of 23 buccal mucosal squamous cell carcinoma (BMSCC)/14 tongue squamous cell carcinoma (TSCC) patients and 30 oral cancer patients from The Cancer Genome Atlas (TCGA) database compared with those in CTAN tissues. Immunohistochemical results showed that protein expression levels of GBP5 were also higher in the tumor tissues of 353 OSCC patients including 117 BMSCC, 187 TSCC, and 49 lip squamous cell carcinoma patients. Moreover, TCGA database analysis indicated that high gene expression levels of GBP5 were associated with poor overall survival in oral cancer patients with moderate/poor cell differentiation, and associated with poor disease-free survival in oral cancer patients with moderate/poor cell differentiation and lymph node metastasis. Furthermore, GBP5-knockdowned cells exhibited decreased cell growth, arrest at G1 phase, and decreased invasion/migration. The gene expression of markers for epithelial−mesenchymal transition and cancer stemness was also reduced in GBP5-silenced oral cancer cells. Taken together, GBP5 might be a potential biomarker and therapeutic target for OSCC patients, especially for those with poor cell differentiation and lymph node metastasis.

## 1. Introduction

Oral squamous cell carcinoma (OSCC) accounts for 90% of all head and neck squamous cell carcinoma (HNSCC) [1] and covers three major histological regions, including buccal mucosal squamous cell carcinoma (BMSCC), tongue squamous cell carcinoma (TSCC), and lip squamous cell carcinoma (LSCC). Although a variety of targeted therapeutic drugs have been tested in OSCC patients [2], the survival rate is still around 50% [3], and nearly up to 40% of OSCC patients subsequently develop recurrences or distant metastases [4]. Thus, identification of more effective and available biomarkers and therapeutic targets for OSCC patients is an unmet need.

The guanylate binding proteins (GBPs) are a family of large interferon-induced GTPases [5] that hydrolyze guanosine triphosphate (GTP). There are seven highly homologous members of GBP in humans, termed HuGBP-1 to HuGBP-7 [6]. GBPs have many cell biologic functions such as antiviral activity [7] and antibacterial infection [8]. Aside from the role GBPs play in pathogen defense, an increasing number of studies have focused on investigating the role of GBPs in cancer development and progression. For example, increased levels of GBP5 expression were found in tumor tissues compared with those in normal tissues, as a higher GBP5 mRNA level is not associated with overall survival and relapse free survival in HNSCC [6]. Nevertheless, the role of GBP5 in OSCC is still unclear.

High-throughput profiling techniques such as RNA-sequencing (RNA-seq) using next-generation sequencing (NGS) [9] and RNAi high-throughput screening (HTS) [10] have been used to identify gene expression associated with many diseases such as cancer, and could provide a very powerful tool for discovering cancer biomarkers and therapeutic targets, especially for OSCC. For example, we have identified GBP6 as a favorable cancer biomarker in TSCC by NGS [11].

In the present study, we found GBP5 to be highly expressed in tumor tissues by comparing the transcriptome profiles of the primary tumor and adjacent normal tissues from two paired OSCC tissues with NGS. Gene expression levels of GBP5 were higher in tumor tissues in our OSCC patients and in oral cancer patients from The Cancer Genome Atlas (TCGA) database. Moreover, high gene expression levels of GBP5 were associated with poor prognosis in oral cancer patients having poor cell differentiation and lymph node metastasis. Furthermore, GBP5-knockdown OSCC cells showed decreased cell growth, G_1_/S arrest, decreased invasion/migration, and cancer stemness. Our findings present the first indication of the clinical significance and biological roles of GBP5 in OSCC.

## 2. Materials and Methods

### 2.1. Patients and Tissue Specimens

All tissue specimens of OSCC patients were obtained from the Department of Pathology at Kaohsiung Veterans General Hospital (VGHKS) between 1993 and 2006, and the study was approved by the VGHKS Institutional Review Board (VGHKS11-CT12-13). A total of 499 margin-free (margin-size ≥ 0.2 cm) paraffin-embedded materials from TSCC (*n* = 245), BMSCC (*n* = 182) patients, and lip SCC (*n* = 72) were established. Patient survival times were recorded from the time of operation to October 2012. The 2002 American Joint Committee on Cancer (AJCC) system was used for pathologic TNM classification.

### 2.2. Laser Capture Microdissection (LCM) and Next Generation Sequencing (NGS) of LCM-Captured Cells

The fresh-frozen tissue samples from two pairs of OSCC patients were used for LCM with the MMI CellCut Plus system. The LCM-captured cells for microdissection were transferred to an miRNeasy Micro Kit (Qiagen GmbH, Hilden, Nordrhein-Westfalen, Germany) for RNA extraction. The extracted RNA samples were then subjected to NGS analysis. More detailed descriptions about LCM and NGS analysis are available in our previous study [11].

### 2.3. Tissue Microarray (TMA) Construction and Immunohistochemistry (IHC)

A TMA block consists of cores constructed from the tumor tissues and corresponding tumor adjacent normal tissues (CTAN), as well as normal uvula epithelium of OSCC patients. All TMA blocks were cut into 4 μm paraffin sections after excluding the cores with incorrect contents. IHC was performed using Novo-Link Max Polymer Detection System (Novocastra Laboratories Ltd., Newcastle Upon Tyne, UK) with a diluted anti-GBP5 monoclonal antibody (dilution 1:250; ProteinTech Group, Inc., Rosemont, IL, USA). Scores for cytoplasmic GBP5 staining were determined by calculating intensity (−, negative; +, weak; ++, moderate; and +++, strong in Figure S1) and percentage (0% to 100%) of positive stained cells. More detailed descriptions of TMA construction, IHC and IHC scoring are available in our previous studies [12,13].

### 2.4. Cell Culture

Three OSCC cell lines from human squamous cell carcinoma of tongue (SAS) and buccal mucosa (TW1.5 and TW2.6) were cultured in DMEM/F12 medium (containing 10% FBS, 100 μg/mL streptomycin, 100 U/mL penicillin, and 1% L-glutamine) (Gibco, Invitrogen Corporation, Carlsbad, CA, USA) and maintained in an incubator with 5% CO_2_ at 37 °C.

### 2.5. Transient and Stable Transfection

For GBP5 transient transfection, OSCC cells were transfected with 10 nM scrambled siRNA or siRNA against GBP5 (Invitrogen Life Technologies, Carlsbad, CA, USA) using RNAiMax (Invitrogen Life Technologies, Carlsbad, CA, USA) for 72 h. For GBP5 stable transfection, HEK293T cells were initially transfected with scramble short hairpin RNA (shRNA) or shRNA against GBP5 using Lipofectamine 2000 (Invitrogen Life Technologies, Carlsbad, CA, USA) for 48 h for amplification of lentivirus particles. After harvesting supernatant containing lentivirus from HEK293T cells, TW1.5 cells were infected with the lentivirus and stable GBP5-silenced TW1.5 cells were selected by antibiotics. More detailed descriptions of GBP5 stable transfection procedures were provided in our previous study [14].

### 2.6. Real-Time PCR (RT-PCR)

The TRIzol reagent (Invitrogen Life Technologies, Carlsbad, CA, USA) was used to extract the total RNA of cells. A total of 1 ug total RNA was converted to cDNA using SuperScript II RNase H-Reverse Transcriptase (Invitrogen Life Technologies, Carlsbad, CA, USA). The amount of mRNA was analyzed in StepOnePlus Real Time PCR System (Applied Biosystems, Foster City, CA, USA) with SYBR Green Master Mix (Applied Biosystems, Foster City, CA, USA), using β-actin mRNA as an internal control [15].

### 2.7. Western Blotting 

SDS-PAGE electrophoresis was used to separate proteins from cell lysates, then the gel was transferred onto a nitrocellulose membrane. The membrane was blocked with 5% skim milk and incubated with primary antibodies overnight at 4 °C. After washing, the membrane was incubated with the HRP-labeled secondary antibody. The proteins were detected with an ECL reagent using the ChemiDoc XRS Imaging System (Bio-Rad Laboratories, Irvine, CA, USA) [16].

### 2.8. Clonogenic Assay

The cells (0.5–1 × 10^3^ cells) were cultured in 6-well plates in complete media for 2 weeks with one medium refreshed every 3 days. The cell colonies were fixed and stained with 2% paraformaldehyde and 0.25% crystal violet, respectively, for 30 min. The cells were then rinsed with PBS and water until the background was clean. The colonies were counted and quantified from at least three independent experiments [17].

### 2.9. Tumor Sphere Formation 

The cells (5 × 10^3^ cells/mL) were seeded into a 96-well, clear, round-bottom, ultra-low attachment Microplate (Corning Costar, Cambridge, MA, USA) for 7 days to form spheroid cells [15], and sphere viability was measured by the 3D CellTiter Glo assay (Promega, Madison, WI, USA) [18].

### 2.10. Cell Cycle Analysis 

The 75% ethanol fixed cells were stained with 50 µg/mL propidium iodide (Sigma-Aldrich, St. Louis, MO, USA) and 25 µg/mL RNase A (Sigma-Aldrich, St. Louis, MO, USA) on ice for 30 min. The FACScan analyzer (Becton, Dickinson and Company, Franklin Lakes, NJ, USA) and FlowJo analysis software (Tree Star, Ashland, OR, USA) were used to estimate and analyze proportions of stained cells in different cell cycle phases, respectively. A more detailed description of the cell cycle analysis was provided in our previous study [19].

### 2.11. Cell Invasion and Migration

For cell invasion, the cells (1.5 × 10^5^ cells/300 μL) were suspended with DMEM containing 1% FBS and seeded in 8 μm pores transwell inserts (Greiner Bio-One, Stroud, UK) coated with 0.5% Matrigel. Afterwards, the invasive cells attached to the bottom of the inserts were fixed and stained with 0.1% crystal violet for qualification. For cell migration, the cells (2 × 10^5^ cells) were cultured into the IBIDI Culture-Inserts (IBIDI, Inc., Planegg, Germany) for 24 h within the culture dishes. Subsequently, the plastic inserts were removed, wound healing was observed, and the migration distance was quantified [20].

### 2.12. Statistical Analysis

RNA-sequencing transcriptome data for 303 oral cancer patients were downloaded from the TCGA database (https://cancergenome.nih.gov, accessed on 13 October 2017) and analyzed. SPSS software (version 20.0, IBM-SPSS Inc., Chicago, IL, USA) was used for statistical analysis. Kruskal−Wallis one-way ANOVA test was used to compare the expression level of GBP5 between CTAN/normal and tumor tissues. In TCGA cohorts, the expression of GBP5 or cancer stemness-related markers was dichotomized to low expression and high expression by the operator characteristic curve (ROC) analysis. A Cox proportional hazards model was used to determine statistically significant prognostic factors. A value of *p* < 0.05 was considered significant.

## 3. Results

### 3.1. Expression Levels of GBP5 between Normal Tissues and Tumor Tissues in Oral Cancer Patients 

**Figure 1:**
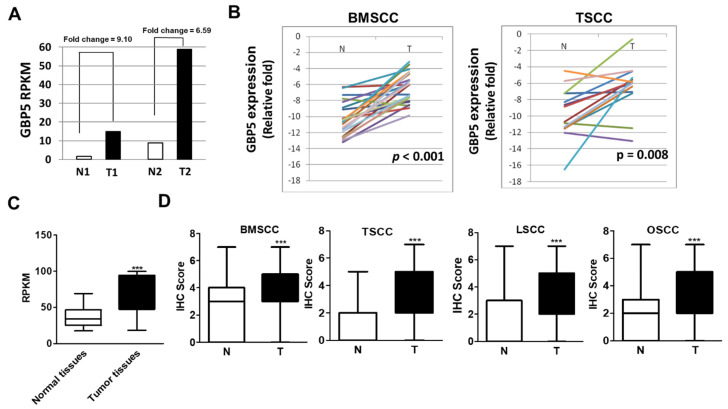
Comparison of GBP5 expression between tumor and adjacent normal tissues in oral cancer patients. (**A**) Relative fold-changes of GBP5 RPKM in CTAN tissues and tumor tissues in two paired OSCC patients by NGS. (**B**) Comparison of GBP5 gene expression between tumor tissues and CTAN tissues from 23 paired BMSCC and 14 paired TSCC patients by RT–PCR. (**C**) RPKM values of GBP5 between 29 normal tissues and 303 tumor tissues in oral cancer patients from TCGA based RNA seq. (**D**) GBP5 IHC scores in CTAN tissues and tumor tissues: 182 BMSCC, 245 TSCC, and 72 LSCC. Data on the whole cohort of 499 patients are also shown (N: CTAN tissues; T: tumor tissues). A value of *p* < 0.05 was considered significant (*** *p* < 0.001).

RNA profiling of CTAN and tumor tissues from two paired OSCC patients at different stages (stages I vs. IV) was performed using NGS. After filtering genes by RPKM and fold change, we found that gene expression levels of GBP5 were increased by 9.10-fold and 6.59-fold in stages I (T1) and stages IV (T2) tumor tissues, respectively, compared with those in CTAN tissues (N1 and N2) (Figure 1A). Subsequently, gene expression levels of GBP5 were validated in normal and tumor tissues for 23 paired BMSCC patients and 14 paired TSCC patients by RT-PCR. Compared with CTAN tissues, the gene expression levels of GBP5 were significantly increased in the tumor tissues of OSCC patients, including BMSCC (*p* < 0.001, Figure 1B) and TSCC (*p* = 0.008, Figure 1B) patients. TCGA data analysis indicated that the gene expression levels of GBP5 in 303 tumor tissues of oral cancer patients were higher than those in 29 normal tissues (*p* < 0.001, Figure 1C). Moreover, the gene expression levels of GBP5 in tumor tissues were higher compared with CTAN tissues in 30 oral cancer patients from the TCGA database (*p* < 0.001, Table S1). 

To further investigate the protein expression level of GBP5 in OSCC, IHC distribution of GBP5 was compared between normal, CTAN, and OSCC tissues (including BMSCC, TSCC, and LSCC). The representative photomicrographs of IHC for GBP5 in tumor tissues are presented as negative (-), weak (+), moderate (++), and strong (+++) in Figure S1A. The protein expression levels of GBP5 in the tumor tissues exceeded that in CTAN from a total of 499 OSCC patients including 182 BMSCC, 245 TSCC, and 72 LSCC patients (Figure 1D, Table 1, Figure S1B). Moreover, GBP5 gradually increased from normal tissues, to CTAN tissues, to tumor tissues in BMSCC and TSCC patients (Table 1). These results indicate that the protein levels of GBP5 were higher in tumor tissues than those in normal tissues of oral cancer patients.

### 3.2. The Association of GBP5 Expression with Prognosis in Oral Cancer Patients

Next, we investigated the prognostic role of GBP5 in oral cancer patients from the TCGA database. We found that high gene expression levels of GBP5 were associated with shorter overall survival (OS) in oral cancer patients with poor cell differentiation (adjusted hazard ratio (AHR) = 1.49, 95% confidence interval (CI) = 1.01–2.21, *p* = 0.047, Table 2) and associated with poor disease-free survival (DFS) in oral cancer patients with poor cell differentiation (AHR = 1.81, CI = 1.03–3.19, *p* = 0.040, Table 3) and lymph node metastasis (AHR = 2.21, CI = 1.09–4.50, *p* = 0.028, Table 3). These results indicate that high gene expression levels of GBP5 were associated with poor prognosis in oral cancer patients.

### 3.3. Role of GBP5 in Cell Growth of OSCC Cells

**Figure 2:**
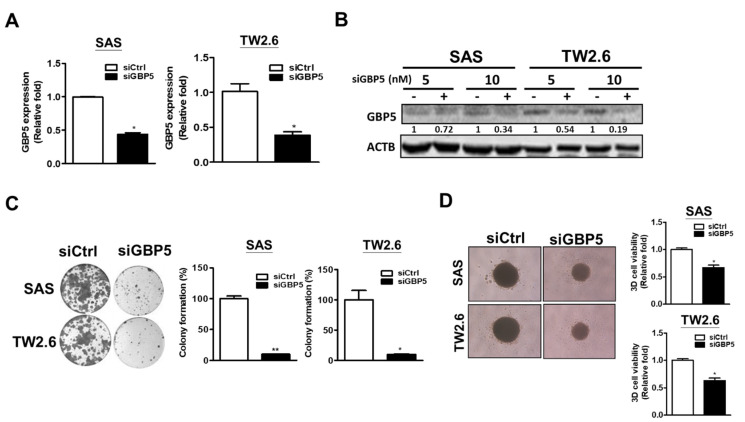
Effects of GBP5 in cell growth of OSCC cells. (**A**) Gene expression levels of GBP5 in transient GBP5–silenced SAS and TW2.6 cells by RT-PCR. All cells were transfected with 10 nM scramble siRNA (siCtrl) or GBP5 siRNA (siGBP5) for 72 h. (**B**) Protein expression levels of GBP5 in transient GBP5–silenced SAS and TW2.6 cells by Western blotting. Beta-actin was used as loading control (ACTB). All cells were transfected with 10 nM scramble siRNA (-) or GBP5 siRNA (+) for 72 h. (**C**) Colony formation of transient GBP5–silenced SAS and TW2.6 cells. After two weeks, these cells were fixed and stained with crystal violet to count the number of colonies. All cells were transfected with 10 nM scramble siRNA (siCtrl) or GBP5 siRNA (siGBP5) for 72 h. (**D**) Formation of tumorspheres and tumorsphere viability of transient GBP5–silenced SAS and TW2.6 cells. Tumorsphere viability was measured by the 3D CellTiter Glo assay. All cells were transfected with 10 nM scramble siRNA (siCtrl) or GBP5 siRNA (siGBP5) for 72 h. All quantitative results are calculated as the mean ± SEM from three independent experiments. A value of *p* < 0.05 was considered significant (** *p* < 0.01, * *p* < 0.05).

To study the role of GBP5 in cancer malignancy in OSCC, the effect of GBP5 in cell growth was investigated in GBP5-knockdown OSCC cells. First, the knockdown efficiency of GBP5 was confirmed by RT-PCR (Figure 2A) and immunoblotting (Figure 2B, Figure S6). As shown, the number of colonies (Figure 2C), the size of tumorspheres, and tumorsphere viability were decreased in GBP5-knockdown OSCC cells (Figure 2D; Figure S2). These results indicate that GBP5 might regulate the growth of OSCC cells.

### 3.4. The Role of GBP5 on Cell Cycle Control in OSCC Cells

**Figure 3:**
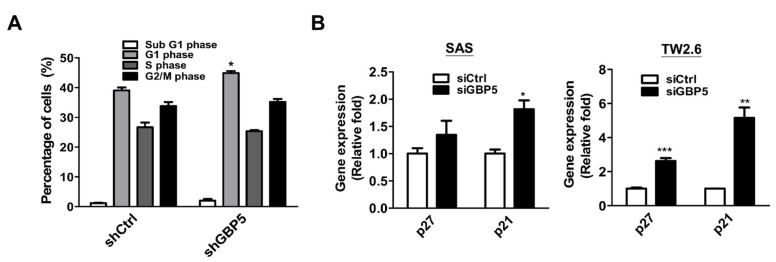
Effects of GBP5 in cell cycle progression of OSCC cells. (**A**) The stable GBP5-silenced TW1.5 cells harboring scrambled shRNA (shCtrl) or shRNA against GBP5 (shGBP5) were seeded for 24 h and harvested for cell cycle distributions by flow cytometry. All fixed cells were stained with propidium iodide to examine the proportions of different cell cycle phases using flow cytometry. FlowJo software was used to analyze the flow cytometry data. (**B**) Gene expression levels of p21 and p27 in GBP5-silenced SAS and TW2.6 cells. All cells were transfected with 10 nM scramble siRNA (siCtrl) and GBP5 siRNA (siGBP5) for 72 h. All quantitative results are calculated as the mean ± SEM from three independent experiments. A value of *p* < 0.05 was considered significant (*** *p* < 0.001, ** *p* < 0.01, * *p* < 0.05).

We found decreased cell growth in GBP5-knockdown OSCC cells (Figure 2). To further investigate whether GBP5 regulates cell growth by controlling the cell cycle, we also evaluated the effect of silencing GBP5 in cell cycle control. We found that stable GBP5-knockdown OSCC cells showed an increased percentage of G_1_ phase cells (Figure 3A), implying that GBP5 might be required for cell cycle regulation, particularly in the transition from G_1_ to S phase of OSCC cells. Moreover, the gene expression levels of two cyclin dependent kinase inhibitors (p21 and p27) were found to be significantly increased in the GBP5-knockdown cells (Figure 3B), supporting the notion that GBP5 induces cell growth by promoting G1/S progression. 

### 3.5. The Role of GBP5 in the Migration and Invasion of OSCC Cells

**Figure 4:**
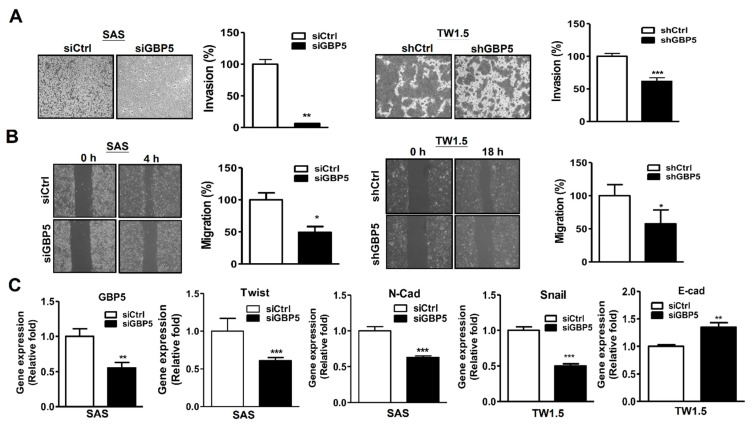
Effects of GBP5 in cell invasion and migration of OSCC cells. (**A**) Invasion of GBP5–silenced SAS and TW1.5 cells. (**B**) Migration of GBP5-silenced SAS and TW1.5 cells. (**C**) Gene expression levels of EMT-related markers in GBP5-silenced SAS and TW1.5 cells by RT-PCR. Transient GBP5–silenced cells were cells transiently transfected with 10 nM scramble siRNA (siCtrl) or GBP5 siRNA (siGBP5) for 72 h. Stable GBP5-silenced cells were cells transfected with scrambled shRNA (shCtrl) or shRNA against GBP5 (shGBP5). All quantitative results are calculated as the mean ± SEM from three independent experiments. A value of *p* < 0.05 was considered significant (*** *p* < 0.001, ** *p* < 0.01, * *p* < 0.05).

Our clinical data indicated that high gene expression levels of GBP5 are associated with shorter DFS in oral cancer patients with lymph node metastasis (Table 3). To investigate the role of GBP5 in the invasion/migration of OSCC cells, the invasion and migration abilities of both transient and stable GBP5-knockdown cells were then evaluated. Our results show that the invasion (Figure 4A) and migration (Figure 4B) abilities in both GBP5-knockdown cells were significantly suppressed compared with those in control cells. Moreover, gene expression levels of epithelial−mesenchymal transition (EMT) markers, such as Twist/N-cadherin and Snail, were decreased in GBP5-knockdown SAS and TW1.5 cells, respectively (Figure 4C). Moreover, the gene expression level of E-cadherin was increased in GBP5-knockdown TW1.5 cells. Protein levels of N-cadherin/Sanil and E-cadherin were also decreased and increased in GBP5-knockdown cells, respectively (Figure S3) These results indicate that GBP5 might be involved in OSCC metastasis via EMT regulation. 

### 3.6. The Role of GBP5 in the Cancer Stemness of OSCC Cells 

**Figure 5:**
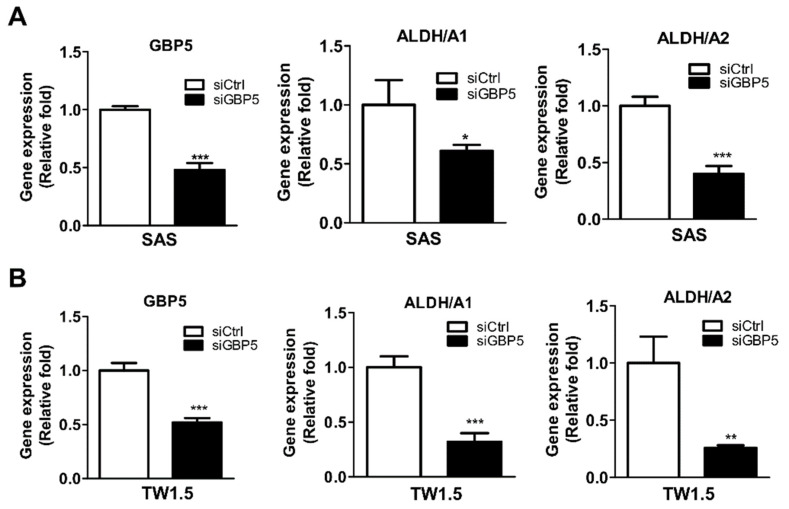
Effects of GBP5 in cancer stemness of OSCC cells. Gene expression levels of cancer stemness-related markers in GBP5-silenced (**A**) SAS and (**B**) TW1.5 cells by RT-PCR. All cells were transiently transfected with 10 nM scramble siRNA (siCtrl) or GBP5 siRNA (siGBP5) for 72 h. All quantitative results are calculated as the mean ± SEM from three independent experiments. A value of *p* < 0.05 was considered significant (*** *p* < 0.001, ** *p* < 0.01, * *p* < 0.05).

Our clinical data showed that high expression levels of GBP5 are associated with DFS (death due to recurrence and any cause) (Table 3). Tumor recurrence might result from those tumors becoming resistant to chemotherapy or radiation therapy [21]. Moreover, cancer stemness contributes to chemotherapy resistance and cancer relapse [22,23]. To investigate the role of GBP5 in OSCC cancer stemness, gene expression levels of several cancer stemness markers such as aldehyde dehydrogenase 1 Family Member A1 (ALDH1A1), ALDH1A2, CD44, CD166, and ABCG2 were analyzed in GBP5-knockdown OSCC cells. Our results indicated that both ALDH1A1 and ALDH1A2 are significantly decreased in GBP5-knockdown SAS (Figure 5A) and TW1.5 cells (Figure 5B). The gene expression levels of CD44, CD166 and ABCG2 were also decreased in GBP5-knockdown SAS and TW1.5 (Figure S4). Additionally, we found that silencing GBP5 may decrease cancer stemness and enhance chemotherapy-induced apoptosis of SAS cells (Figure S5). Moreover, the high co-expressions of GBP5/ALDH1A1 (AHR = 2.63, CI = 1.11–6.19, *p* = 0.027, Table 4), GBP5/ALDH1A2 (AHR = 2.40, CI = 1.09–5.32, *p* = 0.03, Table 4), GBP5/CD166 (AHR = 2.30, CI = 1.07–4.92, *p* = 0.032, Table 4), and GBP5/ ABCG2 (AHR = 2.12, CI = 1.21–3.70, *p* = 0.008, Table 4) genes are found to be associated with OS in oral cancer patients from TCGA database. Furthermore, co-expressions of GBP5/CD166 (AHR = 2.48, CI = 1.08–5.74, *p* = 0.033, Table 5) and GBP5/ ABCG2 (AHR = 2.65, CI = 1.25–5.61, *p* = 0.011, Table 5) genes are associated with DFS in oral cancer patients from TCGA database. These results indicate that GBP5 might be involved in OSCC cancer stemness.

## 4. Discussion

GBP5, belonging to the family of interferon-γ-inducible large GTPases, is one member of seven highly homologous GBPs, and is well known for its role in host defense against pathogens [24]. Nevertheless, the role of GBP5 in cancers, especially in OSCC, is still unknown. In the present study, we first indicated the role of GBP5 in progression and malignancy of OSCC as follows: (1) the expression level of GBP5 is higher in tumor tissues compared with that in normal tissues of oral cancer patients; (2) the high gene expression level of GBP5 is associated with poor prognosis in oral cancer patients with poor cell differentiation and lymph node metastasis; and (3) GBP5 plays a role in cell growth, the cell cycle, the invasion/migration, and cancer stemness of OSCC cells. 

GBPs are known for their function in the defense against bacteria, and numerous studies have documented their oncogenic or tumor suppressive roles for various types of cancer. GBP1 overexpression has been reported to play the oncogenic role and predicts a poor prognosis in glioblastoma, breast cancer, HNSCC [6], esophageal SCC (ESCC) [25], OSCC [26], ovarian cancer [27,28], and prostate cancer [29]. GBP2 expression is significantly higher in ESCC [30]. Higher expressions of GBP2 and GBP3 are significantly associated with shorter relapse-free survival of HNSCC patients [6]. 

In contrast, some GBPs showed anti-tumor effects. For example, GBP1 expression was downregulated and acts as a tumor suppressor in colon cancer [31]. The expression level of GBP2 was higher in breast cancer, but high GBP2 expression is associated with better prognosis in breast cancer patients [32]. Moreover, higher expressions of GBP2, GBP4, and GBP7 are significantly associated with longer OS in HNSCC patients [6]. Moreover, higher GBP1-6 expression is associated with better OS in skin cutaneous melanoma patients [33]. Furthermore, the gene expression level of GBP6 is significantly lower in OSCC patients with poor differentiation/lymph node metastasis, and low expression of GBP6 is associated with poor prognosis in TSCC patients [11]. Regarding current studies of GBP5 in cancers, a truncated splice variant of GBP5 was found in lymphoma [34]. GBP5 is expressed highly in gastric adenocarcinomas as an immune modulator [35]. Moreover, the expression levels of GBP5 are higher in HNSCC tissues [6]. This study found that the high expression levels of GBP5 are associated with cancer malignancy and poor prognosis in oral cancer patients with poor cell differentiation and lymph node metastasis, indicating that GBP5 might play an oncogenic role in OSCC. 

Our results indicated that GBP5 is involved in the cell growth and invasion/migration of OSCC. Epidermal growth factor receptor (EGFR) signaling is known to be an important intracellular signal for cell proliferation [36] and invasion [37]. The knockdown of GBP1 inhibits xenograft growth and the protein expression level of EGFR is decreased in GBP1-knockout xenograft tumors in prostate cancer [29]. Moreover, GBP1 is induced by EGFR signaling and promotes invasion because it is required for the EGF-induction of MMP1 in glioblastoma [37]. GBP1 promotes lymph node metastasis and is positively correlated with EGFR expression in ESCC [27]. The increased expression level of GBP1 was associated with EGFR amplification in glioblastoma multiforme patients with poor prognosis [38], implying that EGFR signaling might also be required for GBP5 induction in OSCC. On the other hand, it is shown that the IFNγ-induced GBP1 is involved in actin remodeling for migration/invasion, which is regulated through the IFNγ/IFN6R/STAT1/IRF2 pathway [39], implying that STAT1 might also be involved in IFNγ-induced GBP5 in OSCC.

Our results indicated that oral cancer patients with high expression levels of GBP5 have poor DFS, which is usually caused by chemotherapy resistance-associated cancer stemness [24]. Here, we found that GBP5 knockdown decreased several chemotherapy resistance-associated cancer stemness markers such as ALDH1A1 [40], ALDH1A2 [41], CD44 [42], CD166 [9], and ABCG2 [43]. In the study, we found that the tumorsphere formation, chemoresistance, and expressions of stemness-related surface markers were all decreased in GBP5-silenced cells, implying that GBP5 expression may directly or indirectly influence the expression levels of genes involved in stem cell physiology. The role of GBP5 on cancer stem cells requires more study to be elucidated.

GBP1 promotes chemoresistance via PGK1-activated EMT signaling in non-small cell lung cancer [44,45]. Moreover, hGBP-1 expression contributes to paclitaxel resistance and causes shorter progression-free survival in ovarian cancers [46]. GBP1 binds to proto-oncogene serine/threonine-protein kinase pim-1 (PIM1) and causes paclitaxel resistance in ovarian cancer [47], implying that PIM1 might interact with GBP5 for drug resistance in OSCC. Further study for the molecular mechanism of GBP5-associated EGFR, STAT1, chemotherapy resistance-associated cancer stemness markers, and PIM1 is warranted in OSCC.

## 5. Conclusions

In conclusion, the high expression levels of GBP5 are associated with poor prognosis in OSCC patients and caner malignancy including cell growth, invasion/migration, and cancer stemness of OSCC, indicating that GBP5 may be a potential diagnostic biomarker and therapeutic target for OSCC patients in the future.

## Figures and Tables

**Table 1:** The comparison of GBP5 expression in the three different tissues of BMSCC, TSCC, LSCC, and OSCC patients.

Variables	Normal Tissue		Tumor Adjacent Normal		Tumor		χ^2^	*p*-Value *
	Mean ± SD	Median	Mean ± SD	Median	Mean ± SD	Median		
BMSCC	(*n* = 30)		(*n* = 117)		(*n* = 182)			
	2.33 ± 2.09	2.00	2.50 ± 1.95	3.00	3.73 ± 1.97	4.00	28.233	<0.001
TSCC	(*n* = 31)		(*n* = 187)		(*n* = 245)			
	1.00 ± 1.41	0.00	1.07 ± 1.51	0.00	3.21 ± 1.90	3.00	138.654	<0.001
LSCC	(*n* = 15)		(*n* = 49)		(*n* = 72)			
	2.33 ± 1.54	2.00	1.53 ± 1.85	0.00	3.68 ± 1.91	4.00	32.544	<0.001
OSCC	(*n* = 76)		(*n* = 353)		(*n* = 499)			
	1.79 ± 1.84	2.00	1.61 ± 1.83	2.00	3.47 ± 1.94	3.00	177.689	<0.001

Abbreviations: BMSCC, buccal mucosal squamous cell carcinoma; TSCC, tongue squamous cell carcinoma; LSCC, lip squamous cell carcinoma; OSCC, oral squamous cell carcinoma; SD, standard deviation. * *p*-values were estimated by Kruskal−Wallis one-way ANOVA test. Bold values denote statistical significance.

**Table 2:** The GBP5 gene expression in overall survival of oral cancer patients from TCGA database.

Variable		No. (%)	CHR (95% CI)	*p-*Value *	AHR (95% CI)	*p*-Value ^†^
Sex						
Female	Low	39 (47.6)	1.00		1.00	
	High	43 (52.4)	1.00 (0.51–1.94)	0.994	1.25 (0.63–2.48)	0.520 ^a^
Male	Low	110 (60.4)	1.00		1.00	
	High	72 (39.6)	1.39 (0.89–2.16)	0.147	1.49 (0.95–2.33)	0.080 ^a^
Age, years						
≤60	Low	73 (65.8)	1.00		1.00	
	High	38 (34.2)	1.46 (0.81–2.63)	0.206	1.60 (0.89–2.90)	0.119 ^a^
>60	Low	76 (49.7)	1.00		1.00	
	High	77 (50.3)	1.06(0.66–1.71)	0.809	1.19 (0.73–1.93)	0.480 ^a^
Cell differentiation						
Well	Low	19 (50.0)	>1.00		1.00	
	High	19 (50.0)	1.00 (0.32–3.13)	1.000	0.74 (0.22–2.51)	0.624 ^b^
Moderate, poor	>Low	130 (57.5)	n">1.00		1.00	
	High	96 (42.5)	1.29 (0.87–1.90)	0.201	1.49 (1.01–2.21)	0.047 ^b^
AJCC pathological stage						
I, II	>Low	>26 (45.6)	1.00		1.00	
	High	31 (54.4)	2.82 (0.72–11.01)	0.136	2.80 (0.71–11.05)	0.141 ^c^
III, IV	Low	123 (59.4)	1.00		1.00	
	High	84 (40.6)	1.23 (0.84–1.82)	0.293	1.31 (0.88–1.94)	0.179 ^c^
T classification						
T1, T2	Low	57 (54.8)	1.00		1.00	
	High	47 (45.2)	1.43 (0.65–3.14)	0.372	1.67 (0.75–3.74)	0.212 ^d^
T3, T4	Low	92 (57.5)	1.00		1.00	
	High	68 (42.5)	1.27 (0.83–1.94)	0.266	1.31 (0.86–2.01)	0.209 ^d^
N classification						
N0	Low	63 (53.4)	1.00		1.00	
	High	55 (46.6)	1.29 (0.68–2.43)	0.442	1.39 (0.73–2.65)	0.314 ^e^
N1, N2, N3	Low	86 (58.9)	1.00		1.00	
	High	60 (41.1)	1.31 (0.83–2.06)	0.241	1.33 (0.84–2.10)	0.225 ^e^
Postoperative RT						
No	Low	54 (54.5)	1.00		1.00	
	High	45 (45.5)	1.24 (0.70–2.22)	0.464	1.61 (0.89–2.92)	0.118 ^a^
Yes	Low	78 (56.1)	1.00		1.00	
	High	61 (43.9)	1.25 (0.72–2.19)	0.430	1.35 (0.77–2.37)	0.293 ^a^

Abbreviations: CHR, crude hazard ratio; CI, confidence interval; AHR, adjusted hazard ratio; AJCC, American Joint Committee on Cancer; RT, radiotherapy. * *p*-values were estimated by Cox’s regression. ^†^ *p-*values were estimated by multivariate Cox’s regression. ^a^ Adjusted for cell differentiation (moderate + poor vs. well) and AJCC pathological stage (stage III+ IV vs. stage I + II). ^b^ Adjusted for AJCC pathological stage (stage III + IV vs. stage I + II). ^c^ Adjusted for cell differentiation (moderate + poor vs. well). ^d^ Adjusted for cell differentiation (moderate + poor vs. well) and N classification (N1, N2, N3 vs. N0). ^e^ Adjusted for cell differentiation (moderate + poor vs. well) and T classification (T3, T4 vs. T1 + T2).

**Table 3:** The GBP5 gene expression in disease-free survival of oral cancer patients from TCGA database.

Variable		No. (%)	CHR (95% CI)	*p*-Value *	AHR (95% CI)	*p-*Value ^†^
Sex						
Female	Low	31 (42.5)	1.00		1.00	
	High	42 (57.5)	1.62 (0.54–4.85)	0.390	1.75 (0.57–5.36)	0.328 ^a^
Male	Low	88 (57.5)	1.00		1.00	
	High	65 (42.5)	1.73 (0.94–3.20)	0.081	1.83 (0.99–3.40)	0.056 ^a^
Age, years						
≤60	Low	57 (60.6)	1.00		1.00	
	High	37 (39.4)	1.65 (0.69–3.98)	0.261	1.83 (0.76–4.41)	0.177 ^a^
>60	Low	62 (47.0)	1.00		1.00	
	High	70 (53.0)	1.54(0.78–3.04)	0.214	1.64 (0.82–3.26)	0.159 ^a^
Cell differentiation						
Well	Low	17 (48.6)	1.00		1.00	
	High	18 (51.4)	1.69 (0.28–10.13)	0.567	1.08 (0.18–6.57)	0.931 ^b^
Moderate, poor	Low	102 (53.4)	1.00		1.00	
	High	89 (46.6)	1.67 (0.95–2.93)	0.073	1.81 (1.03–3.19)	0.040 ^b^
AJCC pathological stage						
I, II	Low	23 (44.2)	1.00		1.00	
	High	29 (55.8)	59.84 (0.20–17,884.73)	0.159	48.43 (0.19–12,494.06)	0.171 ^c^
III, IV	Low	96 (55.2)	1.00		1.00	
	High	78 (44.8)	1.35 (0.76–2.40)	0.307	1.40 (0.78–2.50)	0.256 ^c^
T classification						
T1, T2	Low	49 (51.0)	1.00		1.00	
	High	47 (49.0)	2.28 (0.85–6.07)	0.101	2.50 (0.92–6.78)	0.072 ^d^
T3, T4	Low	70 (53.8)	1.00		1.00	
	High	60 (46.2)	1.48 (0.77–2.85)	0.240	1.50 (0.77–2.89)	0.231 ^d^
N classification						
N0	Low	55 (51.4)	1.00		1.00	
	High	52 (48.6)	1.26 (0.55–2.86)	0.585	1.36 (0.60–3.12)	0.462 ^e^
N1, N2, N3	Low	64 (53.8)	1.00		1.00	
	High	55 (46.2)	2.00 (0.99–4.06)	0.054	2.21 (1.09–4.50)	0.028 ^e^
Postoperative RT						
No	Low	47 (53.4)	1.00		1.00	
	High	41 (46.6)	2.04 (0.74–5.62)	0.167	2.54 (0.90–7.15)	0.078 ^a^
Yes	Low	67 (52.3)	1.00		1.00	
	High	61 (47.7)	1.52 (0.79–2.92)	0.216	1.57 (0.81–3.04)	0.180 ^a^

Abbreviations: CHR, crude hazard ratio; CI, confidence interval; AHR, adjusted hazard ratio; AJCC, American Joint Committee on Cancer; RT, radiotherapy. * *p*-values were estimated by Cox’s regression. ^†^ *p*-values were estimated by multivariate Cox’s regression. ^a^ Adjusted for cell differentiation (moderate + poor vs. well) and AJCC pathological stage (stage III + IV vs. stage I + II). ^b^ Adjusted for AJCC pathological stage (stage III + IV vs. stage I + II). ^c^ Adjusted for cell differentiation (moderate + poor vs. well). ^d^ Adjusted for cell differentiation (moderate + poor vs. well) and N classification (N1, N2, N3 vs N0). ^e^ Adjusted for cell differentiation (moderate + poor vs. well) and T classification (T3, T4 vs T1 + T2).

**Table 4:** The co-expression of GBP5 and cancer stemness markers in overall survival of oral cancer patients from TCGA database.

Variable		No. (%)	CHR (95% CI)	*p-*Value	AHR (95% CI)	*p-*Value
GBP5	Low	149 (56.4)	1.00		1.00	
	High	115 (43.6)	1.23 (0.85–1.77)	0.280 ^a^	1.39 (0.96–2.02)	0.084 ^b^
ALDH1A1	Low	227 (86.0)	1.00		1.00	
	High	37 (14.0)	1.61 (1.03–2.53)	0.038 ^a^	1.33 (0.84–2.10)	0.225 ^b^
ALDH1A2	Low	55 (20.8)	1.00		1.00	
	High	209 (79.2)	2.06 (1.15–3.66)	0.015 ^a^	2.04 (1.14–3.64)	0.016 ^b^
CD166	Low	222 (84.1)	1.00		1.00	
	High	42 (15.9)	1.89 (1.23–2.90)	0.004 ^a^	1.89 (1.23–2.90)	0.004 ^b^
ABCG2	Low	187 (70.8)	1.00		1.00	
	High	77 (29.2)	1.60 (1.09–2.35)	0.016 ^a^	1.38 (0.93–2.04)	0.106 ^b^
GBP5 (L), ALDH1A1 (L)		120 (45.5)	1.00		1.00	
GBP5 (H), ALDH1A1 (L)		107 (40.5)	1.10 (0.76–1.59)	0.624 ^a^	1.32 (0.87–2.00)	0.190 ^c^
GBP5 (L), ALDH1A1 (H)		29 (11.0)	1.42 (0.85–2.34)	0.178 ^a^	1.69 (0.97–2.93)	0.065 ^c^
GBP5 (H), ALDH1A1(H)		8 (3.0)	2.17 (0.95–4.95)	0.066 ^a^	2.63 (1.11–6.19)	0.027 ^c^
GBP5 (L), ALDH1A2 (L)		30 (11.4)	1.00		1.00	
GBP5 (H), ALDH1A2 (L)		25 (9.5)	0.53 (0.24–1.22)	0.139 ^a^	1.06 (0.35–3.15)	0.922 ^c^
GBP5 (L), ALDH1A2 (H)		119 (45.1)	1.02 (0.70–1.47)	0.934 ^a^	1.90 (0.86–4.17)	0.112 ^c^
GBP5 (H), ALDH1A2(H)		90 (34.1)	1.48 (1.02–2.15)	0.040 ^a^	2.40 (1.09–5.32)	0.030 ^c^
GBP5 (L), CD166 (L)		120 (45.5)	1.00		1.00	
GBP5 (H), CD166 (L)		102 (38.6)	1.09 (0.75–1.59)	0.643 ^a^	1.42 (0.93–2.17)	0.108 ^c^
GBP5 (L), CD166 (H)		29 (11.0)	1.80 (1.11–2.92)	0.018 ^a^	2.23 (1.30–3.82)	0.004 ^c^
GBP5 (H), CD166 (H)		13 (4.9)	1.76 (0.86–3.62)	0.125 ^a^	2.30 (1.07–4.92)	0.032 ^c^
GBP5 (L), ABCG2 (L)		104 (39.4)	1.00		1.00	
GBP5 (H), ABCG2 (L)		83 (31.4)	0.93 (0.62–1.38)	0.700 ^a^	1.19 (0.75–1.90)	0.456 ^c^
GBP5 (L), ABCG2 (H)		45 (17.0)	1.25 (0.79–1.98)	0.335 ^a^	1.52 (0.90–2.56)	0.117 ^c^
GBP5 (H), ABCG2(H)		32 (12.1)	1.82 (1.11–2.98)	0.019 ^a^	2.12 (1.21–3.70)	0.008 ^c^

Abbreviations: CHR, crude hazard ratio; CI, confidence interval; AHR, adjusted hazard ratio; L, low expression; H, high expression. ^a^ *p*-values were estimated by Cox’s regression. ^b^ *p*-values were adjusted for cell differentiation (moderate + poor vs. well) and AJCC pathological stage (stage III + IV vs. stage I + II) by multivariate Cox’s regression. ^c^ *p*-values were estimated by multivariate Cox’s regression.

**Table 5:** The co-expression of GBP5 and cancer stemness markers in disease-free survival of oral cancer patients from TCGA database.

Variable		No. (%)	CHR (95% CI)	*p-*Value	AHR (95% CI)	*p-*Value
GBP5	Low	119 (52.7)	1.00		1.00	
	High	107 (47.3)	1.63 (0.95–2.78)	0.074 ^a^	1.78 (1.04–3.05)	0.037 ^b^
ALDH1A1	Low	212 (93.8)	1.00		1.00	
	High	14 (6.2)	1.95 (0.83–4.56)	0.123 ^a^	1.74 (0.74–4.07)	0.205 ^b^
ALDH1A2	Low	198 (87.6)	1.00		1.00	
	High	28 (12.4)	1.16 (0.55–2.46)	0.702 ^a^	1.01 (0.47–2.15)	0.989 ^b^
CD166	Low	148 (65.5)	1.00		1.00	
	High	78 (34.5)	1.45 (0.85–2.47)	0.173 ^a^	1.27 (0.74–2.18)	0.386 ^b^
ABCG2	Low	105 (46.5)	1.00		1.00	
	High	121 (53.5)	1.79 (1.03–3.13)	0.040 ^a^	1.73 (0.99–3.02)	0.054 ^b^
GBP5 (L), ALDH1A1 (L)		108 (47.8)	1.00		1.00	
GBP5 (H), ALDH1A1 (L)		104 (46.0)	1.58 (0.93–2.69)	0.092 ^a^	1.87 (1.05–3.33)	0.034 ^c^
GBP5 (L), ALDH1A1 (H)		11 (4.9)	1.97 (0.79–4.96)	0.148 ^a^	2.79 (1.04–7.51)	0.042 ^c^
GBP5 (H), ALDH1A1(H)		3 (1.3)	1.69 (0.23–12.25)	0.604 ^a^	2.48 (0.33–18.62)	0.376 ^c^
GBP5 (L), ALDH1A2 (L)		106 (46.9)	1.00		1.00	
GBP5 (H), ALDH1A2 (L)		92 (40.7)	1.36 (0.80–2.32)	0.256 ^a^	1.47 (0.83–2.62)	0.185 ^c^
GBP5 (L), ALDH1A2 (H)		13 (5.8)	0.54 (0.13–2.22)	0.390 ^a^	0.68 (0.16–2.90)	0.598 ^c^
GBP5 (H), ALDH1A2(H)		15 (6.6)	1.82 (0.78–4.26)	0.166 ^a^	2.14 (0.87–5.28)	0.100 ^c^
GBP5 (L), CD166 (L)		76 (33.6)	1.00		1.00	
GBP5 (H), CD166 (L)		72 (31.9)	1.32 (0.76–2.29)	0.320 ^a^	2.11 (1.01–4.40)	0.047 ^c^
GBP5 (L), CD166 (H)		43 (19.0)	1.19 (0.64–2.21)	0.594 ^a^	1.99 (0.89–4.45)	0.093 ^c^
GBP5 (H), CD166(H)		35 (15.5)	1.52 (0.79–2.96)	0.212 ^a^	2.48 (1.08–5.74)	0.033 ^c^
GBP5 (L), ABCG2 (L)		55 (24.3)	1.00		1.00	
GBP5 (H), ABCG2 (L)		50 (22.1)	0.68 (0.33–1.39)	0.293 ^a^	1.07 (0.43–2.63)	0.889 ^c^
GBP5 (L), ABCG2 (H)		64 (28.3)	0.82 (0.45–1.51)	0.522 ^a^	1.26 (0.56–2.83)	0.582 ^c^
GBP5 (H), ABCG2(H)		57 (25.2)	2.37 (1.38–4.08)	0.002 ^a^	2.65 (1.25–5.61)	0.011 ^c^

Abbreviations: CHR, crude hazard ratio; CI, confidence interval; AHR, adjusted hazard ratio; L, low expression; H, high expression. ^a^ *p*-values were estimated by Cox’s regression. ^b^ *p*-values were adjusted for cell differentiation (moderate + poor vs. well) and AJCC pathological stage (stage III + IV vs. stage I + II) by multivariate Cox’s regression. ^c^ *p*-values were estimated by multivariate Cox’s regression.

## Data Availability

Not applicable.

## Supplementary Materials

The following are available online at https://www.mdpi.com/article/10.3390/cancers13164043/s1, Figure S1: GBP5 protein levels in tissues of OSCC patients. Figure S2: Formation of tumorspheres and tumorsphere viability of transient GBP5-silenced TW1.5 cells. Figure S3: Protein levels of EMT markers in GBP5-silenced SAS and TW1.5 cells. Figure S4: Gene expression levels of cancer stemness markers in GBP5-silenced SAS and TW1.5 cells. Figure S5: Drug sensitivity in GBP5-silenced SAS cells. Figure S6: Original Western Blots. Table S1: The comparison of GBP5 gene expression between tumor adjacent normal tissues and tumor tissues in oral cancer patients from TCGA database.
