# Supplementary material for: Clinical Significance and the Role of Guanylate-Binding Protein 5 in Oral Squamous Cell Carcinoma
Source: Cancers (Basel). 2021 Aug 11;13(16):4043. doi: 10.3390/cancers13164043 (PMC8394330; doi:10.3390/cancers13164043)
Supplement: Supplementary file 1 [file cancers-13-04043-s001.zip › cancers-1279263-supplementary.pdf]

cancers

Supplementary Materials: Clinical Significance and the Role of Guanylate-Binding Protein 5 in Oral Squamous Cell Carcinoma

Pei-Feng Liu, Chih-Wen Shu, Cheng-Hsin Lee, Huei-Cin Sie, Huei-Han Liou, Jiin-Tsuey Cheng, Luo-Ping Ger, Chun-Lin Chen, Chien-Chou Chen and Chun-Feng Chen

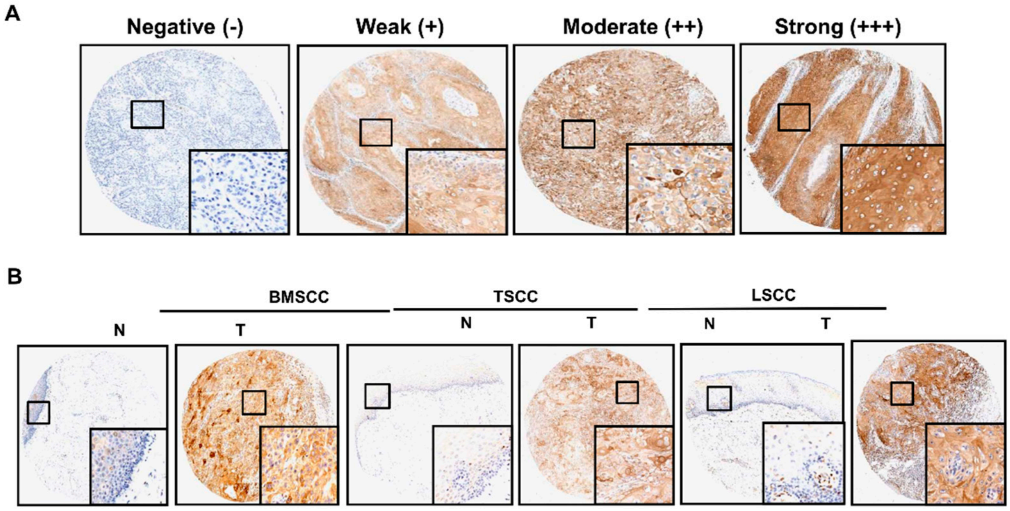

Figure S1. GBP5 protein levels in tissues of OSCC patients. **(A)** Representative IHC images showing intensity scores of GBP5 staining as negative (-), weak (+), moderate (++), and strong (+++) in OSCC patients. **(B)** Representative immunoreactivity intensity of GBP5 between CTAN and tumor tissues of one paired BMSCC, TSCC and LSCC patients.

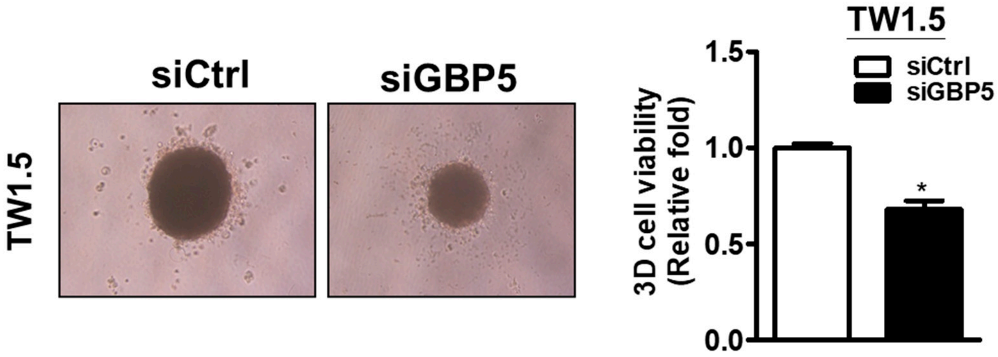

Figure S2. Formation of tumorspheres and tumorsphere viability of transient GBP5-silenced TW1.5 cells. Tumorsphere cell viability was measured by the 3D CellTiter Glo assay. All cells were transfected with 10 nM scramble siRNA (siCtrl) or GBP5 siRNA (siGBP5) for 72h. All quantitative results are calculated as the mean±SEM from three independent experiments.

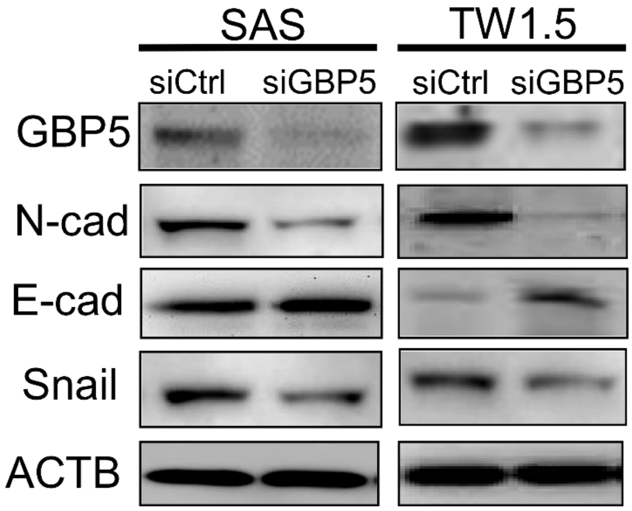

Figure S3. Protein levels of EMT markers in GBP5-silenced SAS and TW1.5 cells. The cells were transiently transfected with 10 nM scramble siRNA (siCtrl) or GBP5 siRNA (siGBP5) for 72h. Protein levels of N-cadherin, E-cadherin and Sanil in GBP5-silenced SAS and TW1.5 cells were analyzed by Western blotting. All quantitative results are expressed as the mean±SEM from three independent experiments.

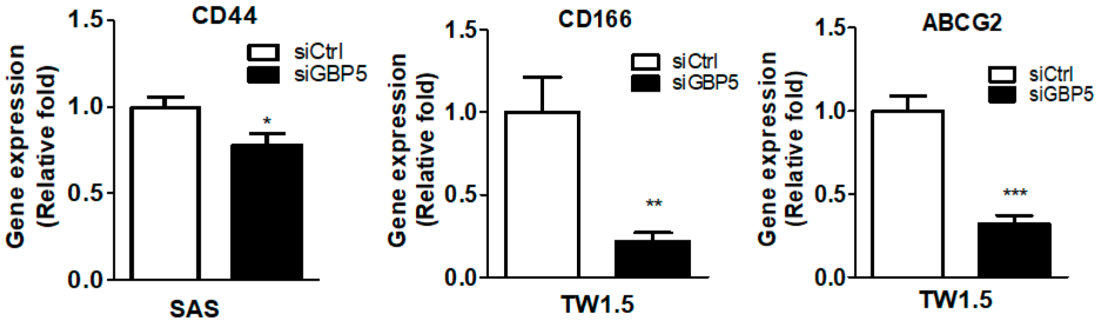

Figure S4. Gene expression levels of cancer stemness markers in GBP5-silenced SAS and TW1.5 cells. All cells were transiently transfected with 10 nM scramble siRNA (siCtrl) or GBP5 siRNA (siGBP5) for 72h. Gene expression levels of CD44, CD166 and ABCG2 in GBP5-silenced SAS and TW1.5 cells were analyzed by RT-PCR. All quantitative results are expressed as the mean±SEM from three independent experiments.

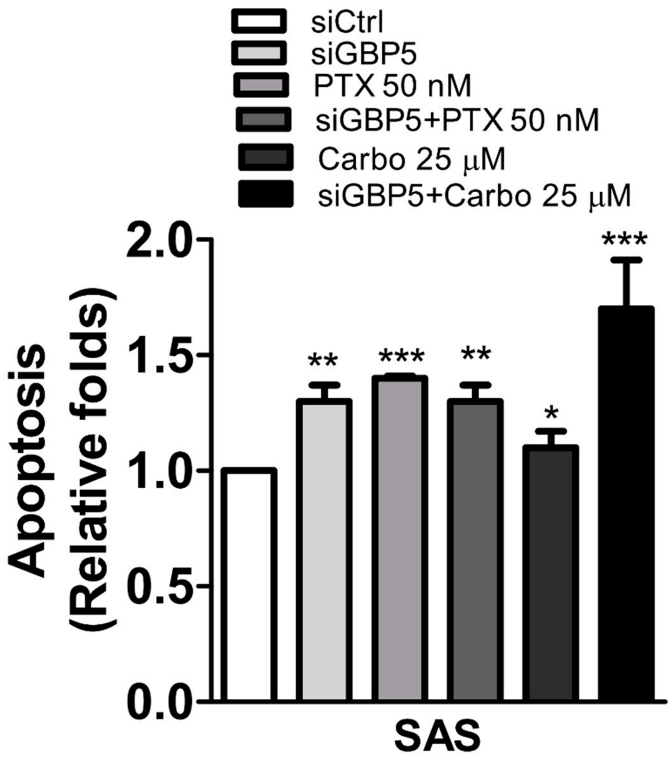

Figure S5. Drug sensitivity in GBP5-silenced SAS cells. SAS cells were transiently transfected with 10 nM scramble siRNA (siCtrl) or GBP5 siRNA (siGBP5) for 48h. Silenced-GBP5 cells were treated with paclitaxel (PTX, 50 nM) or carboplatin (Carbo, 25 μ M) for 24h. Apoptotic silenced-GBP5 treated with paclitaxel (PTX, 50 nM) or carboplatin (Carbo, 25 μ M) were determined with PI/AV staining, followed by analysis with flow cytometry. All quantitative results are expressed as the mean±SEM from three independent experiments.

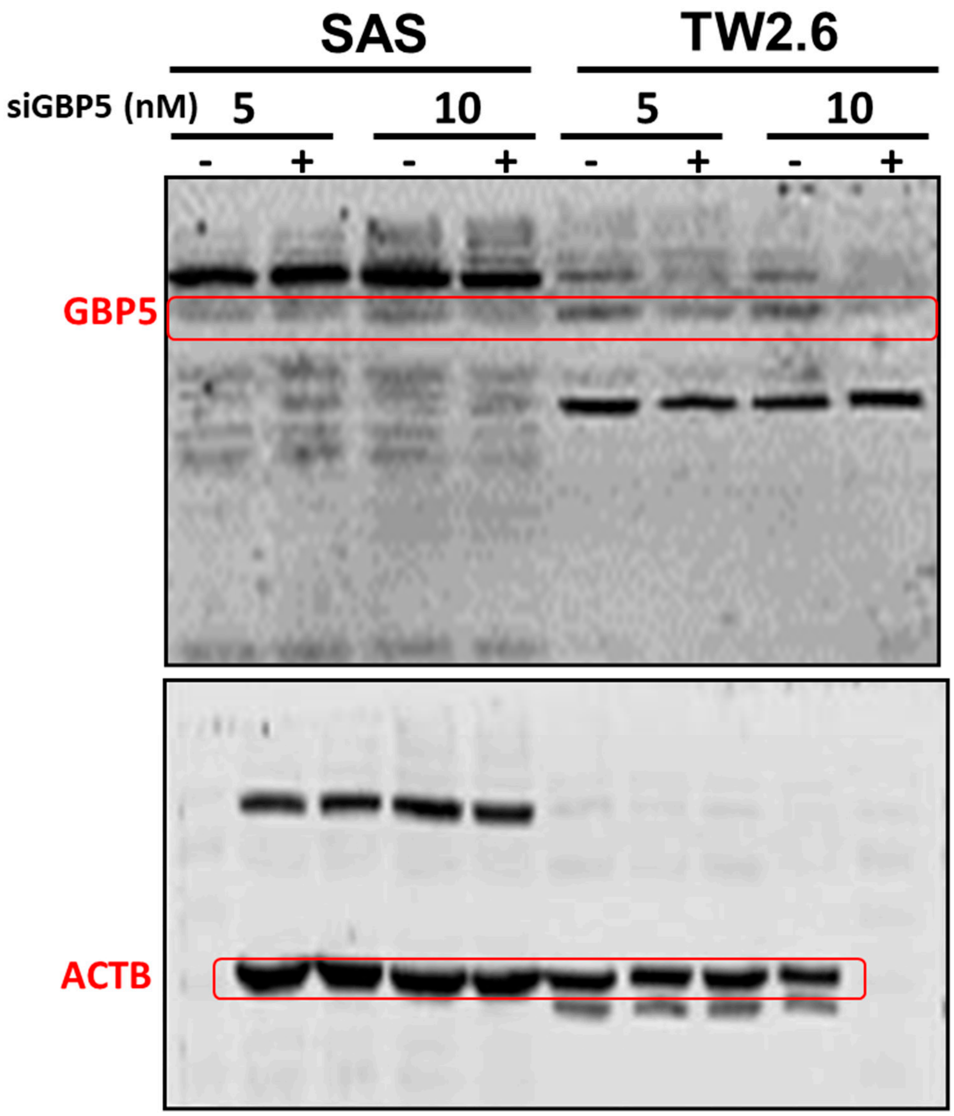

Figure S6. Original Western Blots.

Table S1. The comparison of GBP5 gene expression between tumor adjacent normal tissues and tumor tissues in oral cancer patients from TCGA database.

Variables	No.	Tumor adjacent normal		Tumor		*p*-Value*
		Mean±SD	Median	Mean±SD	Median	
GBP5	30	6.91±1.96	6.6638	9.53±2.16	9.8488	**<0.001**

Abbreviations: SCC, squamous cell carcinoma; SD, standard deviation. **p*-values were estimated by Paired t-test.
